# Demarcation of local neighborhoods to study relations between contextual factors and health

**DOI:** 10.1186/1476-072X-9-34

**Published:** 2010-06-29

**Authors:** Simone M Santos, Dora Chor, Guilherme Loureiro Werneck

**Affiliations:** 1 Health Information Department - LIS/ICICT/FIOCRUZ and Department of Epidemiology and Quantitative Methods - DEMQS/ENSP/FIOCRUZ, Rio de Janeiro, Brazil; 2 Department of Epidemiology and Quantitative Methods (DEMQS/ENSP/FIOCRUZ), Rio de Janeiro, Brazil; 3 Institute of Social Medicine (IMS/UERJ), Rio de Janeiro, Brazil

## Abstract

**Background:**

Several studies have highlighted the importance of collective social factors for population health. One of the major challenges is an adequate definition of the spatial units of analysis which present properties potentially related to the target outcomes. Political and administrative divisions of urban areas are the most commonly used definition, although they suffer limitations in their ability to fully express the neighborhoods as social and spatial units.

**Objective:**

This study presents a proposal for defining the boundaries of local neighborhoods in Rio de Janeiro city. Local neighborhoods are constructed by means of aggregation of contiguous census tracts which are homogeneous regarding socioeconomic indicators.

**Methodology:**

Local neighborhoods were created using the SKATER method (TerraView software). Criteria used for socioeconomic homogeneity were based on four census tract indicators (income, education, persons per household, and percentage of population in the 0-4-year age bracket) considering a minimum population of 5,000 people living in each local neighborhood. The process took into account the geographic boundaries between administrative neighborhoods (a political-administrative division larger than a local neighborhood, but smaller than a borough) and natural geographic barriers.

**Results:**

The original 8,145 census tracts were collapsed into 794 local neighborhoods, distributed along 158 administrative neighborhoods. Local neighborhoods contained a mean of 10 census tracts, and there were an average of five local neighborhoods per administrative neighborhood.

The local neighborhood units demarcated in this study are less socioeconomically heterogeneous than the administrative neighborhoods and provide a means for decreasing the well-known statistical variability of indicators based on census tracts. The local neighborhoods were able to distinguish between different areas within administrative neighborhoods, particularly in relation to squatter settlements.

**Conclusion:**

Although the literature on neighborhood and health is increasing, little attention has been paid to criteria for demarcating neighborhoods. The proposed method is well-structured, available in open-access software, and easily reproducible, so we expect that new experiments will be conducted to evaluate its potential use in other settings. The method is thus a potentially important contribution to research on intra-urban differentials, particularly concerning contextual factors and their implications for different health outcomes.

## Introduction

In the area of epidemiological studies, the 1990s witnessed increasingly widespread use of ecological methods for the study of contextual factors, a field known as ecoepidemiology [1-4]. Since then, various researchers have focused on improving methods that allow a better grasp of the importance of collective social factors in processes related to population health [5-8]. The central concept of this research is that although health outcomes occur in individuals, a large share of the determinants of these processes take place at other levels, referred to generically as collective or contextual [8,9]. The development of multilevel statistical models that allow analysis of contextual levels simultaneously with the individual level has helped expand our understanding of the role played by multiple social factors in health outcomes [10-13].

Although spatial approaches have still not been fully used in epidemiological studies in Brazil, their integration into research on contextual factors shows huge potential for application in health studies. One of the most widely used ways of demarcating population groups on collective scales is by spatial partitioning of the territory [14-17]. Area of residence, for example, has been used to grasp the social and environmental conditions to which these groups are exposed [18].

In addition to improvement in measurement and data sources for intrinsic group-level properties, another major challenge for researchers is the definition of adequate spatial units of analysis for studying properties potentially associated with the target outcomes. Particularly in countries with capitalist economies, and especially in developing countries like Brazil, the way urban territory is occupied both reflects and is conditioned by the political and economic macrostructure. Thus, the incorporation of spatial units in the study of social inequalities in health is essential for capturing these conditioning and determinant factors.

Spatial units of analysis at the contextual level vary according to the scales of investigation (global, regional, local) and criteria (social, political-administrative, ecological) adopted by the study. The most commonly used divisions of municipal urban territory in health studies at the local level are political-administrative, like districts or boroughs, administrative neighborhoods, ZIP areas, and census tracts [19]. Linked to these political-administrative spatial sections, various types of information are available in databases, including Health and Environmental Information Systems [20].

In a recent series of North American and British studies, various political-administrative units of analysis, referred to generically as "neighborhoods" [21], have been used to detect relevant contextual effects in the occurrence of health outcomes, as for example in self-rated health, children's health [22], infectious diseases, adult health [23,24], lifestyle [25,26], mortality [27], and others. Based on the results of these and other studies, contextual socioeconomic factors exert a specific influence on the prediction of health outcomes, even after considering individual socioeconomic conditions [28].

The main advantage of using political-administrative units is the ease in georeferencing various data in geographic information systems (GIS). Having been organized in hierarchically nested subsets, information at both the individual level (whose address should be geocoded) and other levels can be referred to the respective political-administrative unit for study at the collective level. Although such divisions are useful for a general approach, they present problems in relation to their availability and limitation for health research and public policy proposals.

The demarcation of administrative districts and administrative neighborhoods (subdivisions of municipality) is not legally regulated in all Brazilian municipalities, and only some large State capitals have such geographically demarcated units. Even where they exist, these units include populations of widely varying sizes, with highly diverse residential patterns and very heterogeneous socioeconomic levels. Meanwhile, census tracts are minimum spatial units for census data collection and spatial reference [23] but with insufficient size to represent collective social processes that occur at the local level [29,30]. In addition, the small number of inhabitants in census tracts produces problems of excessive statistical variability in the epidemiological and social indicators.

From the point of view of the social unit, few studies have focused on guaranteeing the representation of social processes at the collective level. The concept of neighborhoods as *"distinctive areas into which larger spatial units may be subdivided... The distinctiveness of these areas stems from ... geographical boundaries, ethnic or cultural characteristics of the inhabitants, psychological unity among people who feel that belong together, or concentrated use of an area's facilities for shopping, leisure, and learning" *[31], integrates the sociological approach and provides the basis for the demarcation of representative spatial units for social processes in order to study potentially important contextual factors for health outcomes. In this sense, a proposal that has been explored is the demarcation of local neighborhoods as units of analysis, consisting of sets of relatively homogeneous census tracts according to socioeconomic and spatial contiguity criteria, such as that designed in the Project on Human Development in Chicago Neighborhoods [32].

As a spatial construct, the neighborhood denotes a geographic unit whose residents share proximity and the circumstances that derive from it [33], like social unity, involving recognition of identity among the inhabitants and in the development of interpersonal networks between neighbors. These social properties are important for (1) supporting collective actions in given circumstances and (2) providing the basis and motivation for collective actions [34]. To allow the study of these properties and their influence on health, an operational definition of neighborhood is essential, which can be facilitated by means of GIS tools and the availability of georeferenced social and population data.

In Rio de Janeiro, in particular, where the model of socio-spatial segregation differs from the downtown-versus-suburb pattern [35], administrative neighborhoods are real mosaics that harbor areas of great socioeconomic prosperity, permeated by impoverished areas. Some cases involve islands of prosperity or poverty. To contemplate this complexity, the demarcation of local neighborhoods as units of spatial and social analysis based on the clustering of census tracts is a plausible alternative, since it allows capturing diverse socio-spatial processes that occur among residents of these areas.

The objective of this work is to propose the demarcation of local neighborhoods as geographic units, through spatial analysis that combine contiguous and socio-demographically homogeneous census tracts. The expectation is to discriminate between distinct population groups in the city of Rio de Janeiro.

## Methodology

In 2000, the city of Rio de Janeiro had a total population of some 7,000,000 and consisted of 8,145 census tracts [36] distributed across 158 administrative neighborhoods.

The procedure for the creation of local neighborhoods was based on clustering of census tracts with permanent private households (homes occupied throughout the year, regardless of season), contiguous and internally homogeneous in relation to the selected socioeconomic indicators.

The procedures for classification of areas that allowing clustering a large set of data from smaller areas in groups (regions of analysis) with the objective of maximizing the internal (within-group) homogeneity and external (between-group) heterogeneity are referred to as regionalization. According to Duque et al. [37], various methods can be used for regionalization, and they include two major groups, differentiated on the basis of whether or not they explicitly consider spatial contiguity between areas.

In the current study, among the methods that consider spatial contiguity, we conducted a classification of the census tracts for spatial clustering in local neighborhoods using the SKATER method (*Spatial 'K'luster Analysis by Tree Edge Removal*), using algorithms adapted by Assunção et al. [38], initially proposed for use by the Brazilian Institute of Geography and Statistics (IBGE), or National Census Bureau, and subsequently compiled in the Skater software and made available through TerraView [39]. This method is a heuristic model based on the graph theory [40], whose partitioning is performed with the "spanning tree edge removal" method. SKATER was designed to define homogeneous areas based on clustering of smaller areas (spatial objects) according to control variables (indicators), using the distance between their values as the combination pattern and aiming for the areas to have a minimum, previously stipulated population size. Connectivity graphs are created in order to capture the local neighborhood relationship between spatial objects and summarizes it in a minimum spanning tree whose edges (links) with the highest degree of dissimilarity are pruned successively [41]. The result is the classification of the spatial objects in regions with maximum internal homogeneity. The geographic boundaries of the administrative neighborhoods were respected such that the demarcated local neighborhoods are hierarchically nested subsets within them, that is, there are no local neighborhoods with census tracts that belong to different administrative neighborhoods. In addition, the boundaries imposed by large geographic barriers like highways, railways, lagoons, and islands were also maintained (according with these 'natural' boundaries).

After creation of the local neighborhoods, we identified areas that are not necessarily geographically connected but which display similar socio-demographic characteristics, although they are located in distant administrative neighborhoods, and which we refer to as "super-groups". For this purpose, we conducted an analysis of the clustering of local neighborhoods with homogeneous socio-demographic patterns, using the non-hierarchical K-means method.

The analyses were performed with the SPSS^® ^[42] and TerraView [39] programs and Google Earth™ [43].

### Data sources

• Brazilian population census, 2000;

• Map databases of census tracts and administrative neighborhoods in the city of Rio de Janeiro for the year 2000, from the Health Information Laboratory of the Institute for Scientific and Technological Communication and Information, Oswaldo Cruz Foundation (LIS/ICICT/FIOCRUZ);

• Satellite images available for viewing on Google Earth™ [43].

### Stages performed for demarcation of local neighborhoods

• Creation of socio-demographic indicators based on data from the 2000 population census for the census tracts comprising the city of Rio de Janeiro;

• Exclusion of non-residential census tracts, with no population, with fewer than five permanent private households;

• Cartographic revision of the map database of census tracts and administrative neighborhoods in the city of Rio de Janeiro (i.e. lines of boundaries of some census tracts polygons were not well connected; polygons of lagoons were excluded);

• Linkage of the indicators to the map database;

• Construction of local neighborhoods of census tracts, considering the criteria of contiguity (shared boundaries) and contingency (administrative neighborhood);

• Cluster analysis of homogeneous census tracts (*Cluster SKATER*).

### Criteria for definition of local neighborhoods

#### • Choice of indicators

The choice of socio-demographic indicators considered their relevance and variation across space, in order to allow discriminating between distinct areas according to each variable, and was based on previous studies in which the available variables were selected by means of principal components analysis [41,44,45]. We have also chosen less heavily correlated indicators, since they were more adequate for submitting to cluster analysis, thus avoiding redundancy of information [46]. For example, if we had two indicators that were heavily correlated they could be indicating the same phenomena or processes characterizing redundant information on the dataset. In this case, it is recommended to choose one of them to submit to cluster analysis. In short, the objective was to define the minimum number of variables capable of discriminating between different population profiles.

Indicators were selected from the following three domains.

First, demographic characteristics:

1 - total population that is a key variable to delimit the minimum of population in regionalization process;

2 - permanent private household indicator of demographic concentration;

3 - concentration of children aged 0 through 4 years as a proxy of birth rate that allows the identification of deprived areas (where birth rates were greater than in prosperous areas);

4 - economic dependency ratio that configures an index of people outside the workforce, the dependent population;

5 - male/female ratio that is important to describe the demographic composition of areas.

Second, housing conditions:

1 - inadequate sanitation conditions that allow the distinction of different urban services available at different areas;

2 - concentration of rented homes, a typical pattern of Brazilian middle class, distinguishing them from areas where home ownerships are more common (more frequent in slums and prosperous areas);

3 - concentration of houses (not apartments) as opposed to vertical expansion that indicates the dominant settlement characteristic of urban sets, usually present in areas with high demographic concentration;

4 - inhabitants per household to identify crowded households (the information of the number of inhabitants per room is no more available in Brazil because there were changes in last census methodology).

Third, household conditions:

1 - mean heads-of-households schooling, a traditional indicator of population socioeconomic status;

2 - mean heads-of-households income, a traditional indicator of population socioeconomic status that discriminate nuances of different areas;

3 - mean heads-of-households income greater than 20 times Brazilian minimum wage, a variant of mean income particularly used to identify prosperous areas in Brazil.

**Figure 1:**
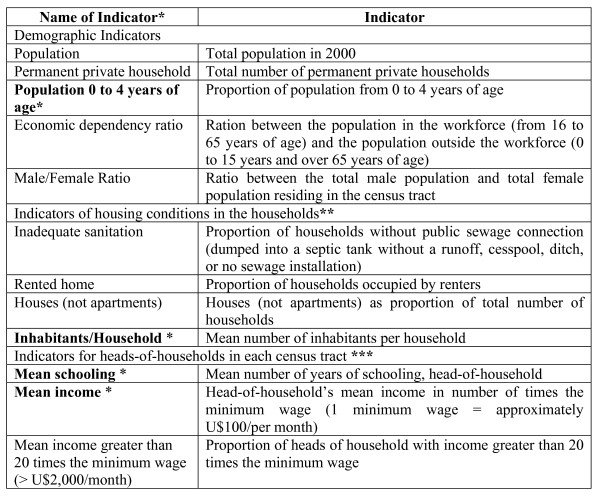
**Chart1. Socioeconomic indicators of census tracts for demarcation of local neighborhoods, city of Rio de Janeiro, Brazil, 2000**. * Indicators used in the final reduced model. ** All the proportions were calculated using the total number of households in the census tract as the denominator. *** The proportions were calculated based on the total number of heads-of-households in the census tract.

Starting with a set of 10 indicators (Figure 1), we did various combinations, thereby reducing the number of indicators to the minimum set that allowed adequate demarcation of local neighborhoods. Most of these indicators has been used by the Brazilian Census Bureau and researchers for describing socio-demographics characteristics of Brazilian urban population. [41,44,45]. The adequacy of boundaries of local neighborhoods obtained with each combination of indicators was analyzed mainly by a visual assessment (as detailed bellow).

All the variables were normalized before classification, not only because some of them did not display normal distribution but also to avoid the influences of the nature of each variable (i.e. some variables were percentages, others ratio-normalization ensures that they "have the same weight" in the classification of cluster analysis).

#### • Population size

After analyzing the mean and maximum population size for spatial units of local neighborhoods obtained from the initial minimum population sizes (of 10,000, 7,500, and 5,000 individuals) and their respective boundaries, we established the minimum population of 5,000 residents to form each local neighborhood.

We avoided obtaining isolated areas with less than the minimum required number of inhabitants. This situation happened with all minimum population sizes because those areas could not be aggregated into a larger cluster group either because they did not show similarity regarding the socioeconomic indicators with a contiguous cluster group or because the generated cluster group should have boundaries falling inside an administrative neighborhood (the contingency geographic unit).

#### • Contingency geographic unit

The boundaries of the administrative neighborhoods were maintained, as a contingency geographic unit for regionalization, to provide the use of one more hierarchical level in future multilevel studies, due to the widespread availability of data in health information systems linked to this unit;

#### • Visual assessment by overlapping layers

The resulting local neighborhood partitions were critically evaluated by means of overlapping layers in a GIS environment and visual observation of the boundaries imposed by major geographic barriers: highways, railways, and natural geographic accidents like massifs, lagoons, and islands.

The polygons in the local neighborhood spatial units were compared visually to the presence of geographic barriers existing in the territory, identified by means of satellite images, so as to ensure that the local neighborhoods did not display such barriers internally but only on their edges (for example, a major avenue should not cross a local neighborhood, since would pose a geographic barrier that "isolates" the resident population along one of its sides from those living on the opposite side). We also visually analyzed the presence of major contrasts displayed in the form of urban occupation with different social patterns inside each administrative neighborhood; together with the production of thematic maps of socioeconomic indicators, this allowed verifying the adequacy of the boundaries for the local neighborhoods created by the process. The choice of a minimum population of 5,000 proved to be the most adequate, since for example other alternatives did not distinguish well between slums (*favelas *or shantytown areas) and the areas surrounding them versus the areas with distinct patterns comprising some administrative neighborhoods.

## Results

### Demarcated local neighborhoods and their socioeconomic profile

The administrative neighborhoods comprising the city of Rio de Janeiro contain populations varying from 136 to 297,459 inhabitants, while the census tracts vary from 136 to 4,529 inhabitants. As a result, the mean number of local neighborhoods per administrative neighborhood was five (median 3.5) ranging from 1 (the minimum in four cases described bellow) to 39 (maximum subdivision) presented at the most populated administrative neighborhood. Larger administrative neighborhoods (more people) tend to present higher socioeconomic variability and were partitioned in more local neighborhoods.

From the total of 158 administrative neighborhoods in the city, four had total population fewer than the minimum population of 5,000 inhabitants and configured geographic isolated areas, with socioeconomic pattern extremely different from their surroundings. Nonetheless, these administrative neighborhoods were classified as four local neighborhood units: 1 - Grumari (a settlement in a coastal natural preservation area); 2 - Joá (a settlement in a coastal rock mountain); 3 - Cidade Universitária (an irregular area inside universitary campus); and 4 - Ilha de Paquetá (an island).

The mean number of census tracts allocated to each local neighborhood was 10, ranging from a minimum of one to a maximum of 36. Besides the exceptions mentioned above, five local neighborhoods with fewer than 5,000 inhabitants were demarcated as a result of the classification method. These clusters were composed by socioeconomically different census tracts compared to their surrounding, but the total population at each local neighborhood reaches slightly more than 4,000 inhabitants.

The mean number of permanent private households per local neighborhood was 2,269 (SD 758.75), ranging from 25 (Joá exception) to 6,072.

The local neighborhoods constructed on the basis of the 10 indicators totaled 800 geographic units, while those demarcated on the basis of four indicators totaled 794, with no important differences in the internal partitioning of the administrative neighborhoods. Thus, we chose the demarcation achieved with the smallest number of indicators for the final model, based on four socioeconomic indicators. These indicators were population 0 to 4 years of age, inhabitants per household, mean schooling and mean income (Figure 1). The Google Earth™ tools for approximating and distancing images, as well as for rotating the point of view and three-dimensional effects, combined with the thematic maps and road maps of the areas comprising the city, allowed evaluating the local neighborhoods' geographic boundaries.

**Figure 2:**
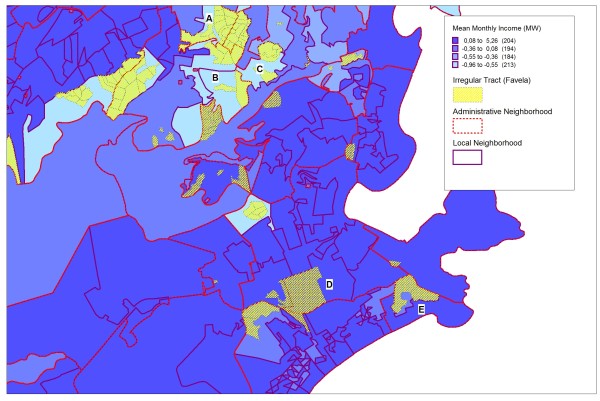
**Local neighborhoods according to mean income categories and distribution of irregular census tracts in an area on the South Side of the city of Rio de Janeiro, Brazil, 2000**.

Figures 2 illustrates the demarcation of local neighborhoods in a selected area of the city's South Side (Zona Sul), highlighting the distribution of *favelas *(hatched areas) in some of the local neighborhoods. For thematic visualization, we used the distribution of standardized mean income categories (values with the mean centered on zero so that negative values are below the mean and positive values above it). We observed two important results that contribute to consider local neighborhoods boundaries adequate. First, we observed that the regular census tracts located around irregular tracts (*favelas*) and with a similar income pattern to them were included in the same local neighborhood as shown in Figure 2 (situations A, B, and C, for example). Second, we observed that irregular tracts (*favelas*) adjacent to regular tracts with much diverse economic pattern were allocated into different local neighborhoods as shown in Figure 2 (situations D and E, for example).

**Figure 3:**
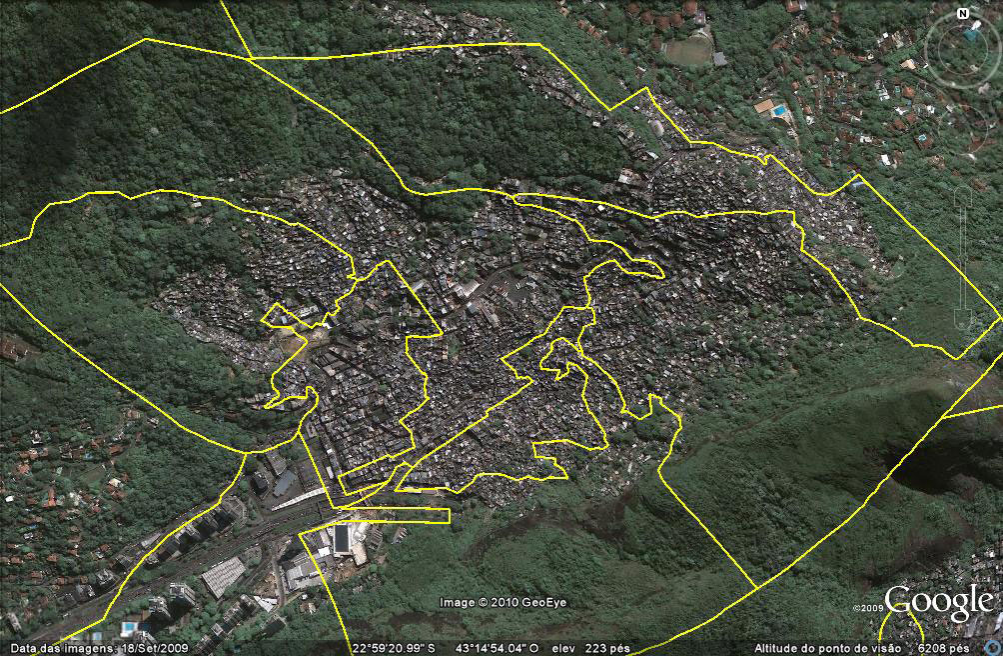
**Boundaries of local neighborhoods (yellow polygons) laid over a satellite image of the Rocinha administrative neighborhood in the city of Rio de Janeiro, Brazil, 2000**.

**Figure 4:**
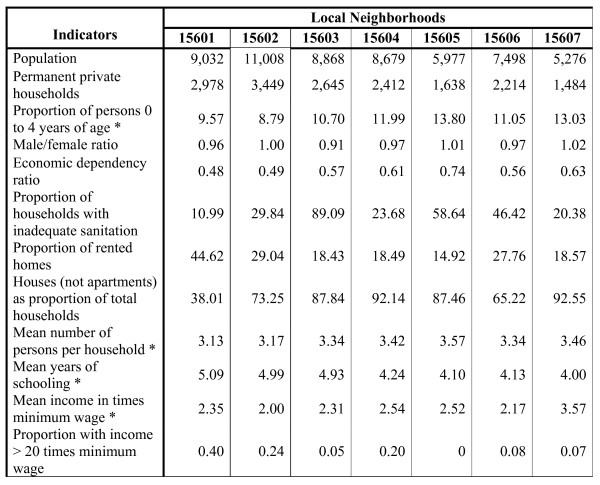
**Chart 2. Socioeconomic characteristics of the seven demarcated local neighborhoods constituting the Rocinha administrative neighborhood, city of Rio de Janeiro, Brazil, 2000**. * Indicators used in the final model.

As shown in Figure 3, the proposed model allowed discriminating between different socioeconomic profiles, even in a set with irregular occupation, as featured in the example by the Rocinha administrative neighborhood, considered homogeneous by the municipal government but not homogeneous by our modeling strategy. Therefore it was divided into seven distinct local neighborhoods (delimited by yellow lines). Figure 4 shows the profile of indicators characterizing the seven local neighborhoods demarcated into Rocinha administrative neighborhood.

**Figure 5:**
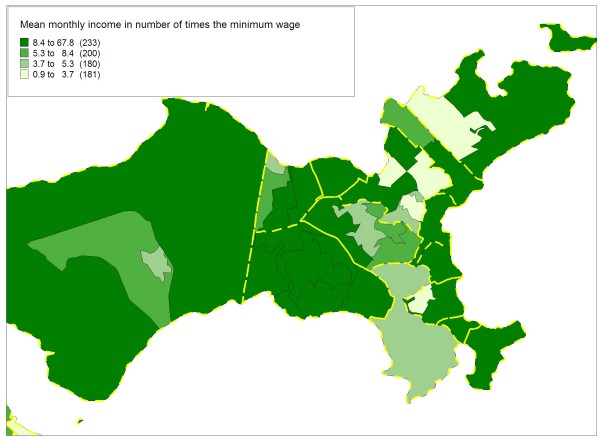
**Mean monthly income in number of times the minimum wage, Ilha do Governador area, Rio de Janeiro, Brazil, 2000**. (1 monthly minimum wage equals approximately U$100).

**Figure 6:**
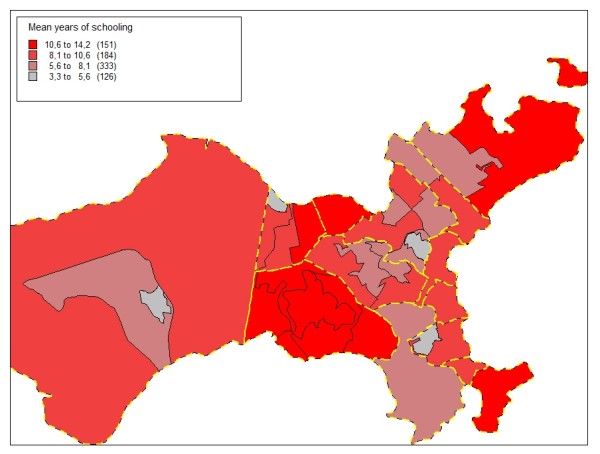
**Mean years of schooling, Ilha do Governador area, Rio de Janeiro, Brazil, 2000**.

**Figure 7:**
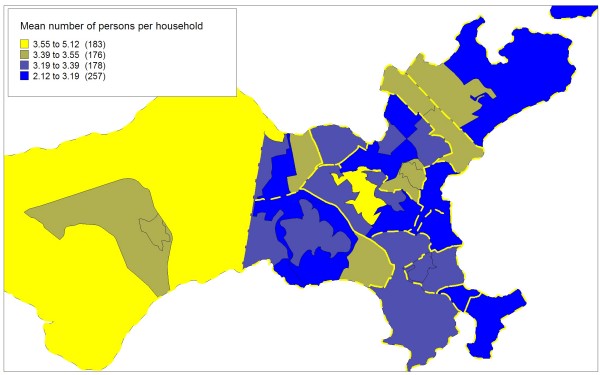
**Mean number of persons per household, Ilha do Governador area, Rio de Janeiro, Brazil, 2000**.

**Figure 8:**
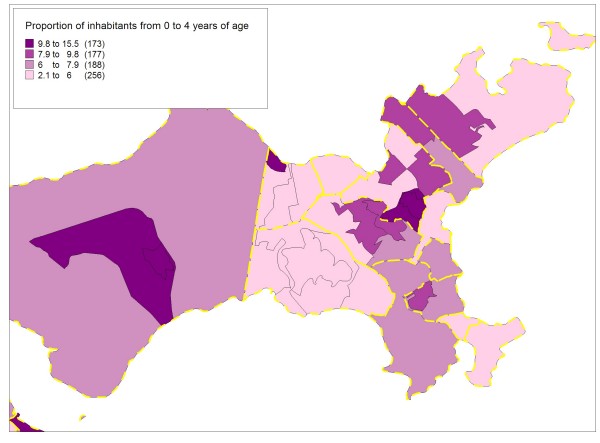
**Proportion of inhabitants from 0 to 4 years of age, Ilha do Governador area, Rio de Janeiro, Brazil, 2000**.

Figures 5, 6, 7 and 8 highlight the Ilha do Governador area and present the contribution of each of the four socioeconomic indicators to the classification and demarcation of local neighborhoods. Within each administrative neighborhood (polygons with thicker lines), demarcation of the local neighborhoods appears (polygons with thinner lines) with the thematic visualization of the distribution (in categories) of the indicators used in the final model: mean monthly income in number of times the minimum wage (figure 5); mean years of schooling (figure 6); mean number of persons per household (figure 7); and proportion of inhabitants from zero to four years of age (figure 8).

### Socioeconomic super-groups

**Figure 9:**
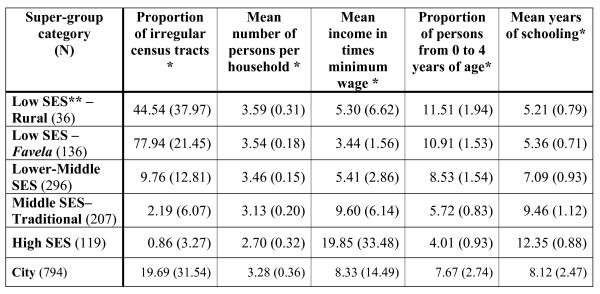
**Chart 3. Socioeconomic characteristics of the super-groups, city of Rio de Janeiro, Brazil, 2000**. * In the cells: mean and standard deviation (parentheses). ** SES - socioeconomic status.

Clustering of local neighborhood sets with similar profiles in terms of socioeconomic status (SES), even though geographically distant, allowed a synthesis of the profile of indicators in five super-groups: 1- low SES with low population density (rural); 2 - low SES with high population density (*favela*); 3 - lower-middle SES; 4 - middle SES; and 5 - high SES (Figure 9).

**Figure 10:**
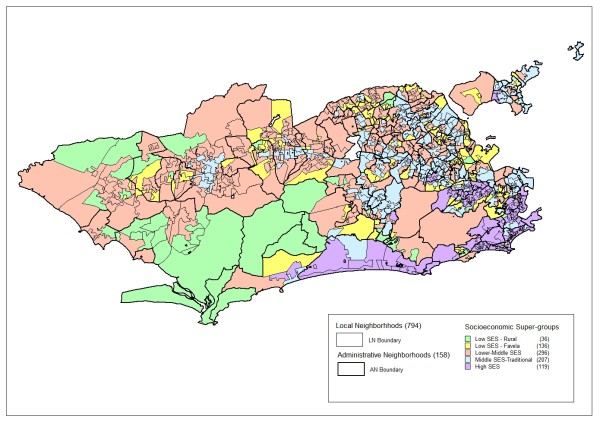
**Spatial distribution of demarcated local neighborhoods, boundaries of administrative neighborhoods, and socioeconomic super-groups, city of Rio de Janeiro, Brazil, 2000**.

Figure 10 allows visualizing the spatial distribution of local neighborhoods resulting from the proposed method (polygons with thinner lines) and their inclusion in the socioeconomic super-groups (visualization theme). It is thereby possible to characterize the sets of local neighborhoods comprising the city of Rio de Janeiro.

## Discussion

Given the relatively large size of the administrative neighborhoods, they tend to show sharp socio-demographic heterogeneity. The local neighborhood units demarcated by this study allowed decreasing this residential and socioeconomic heterogeneity, adequately separating the distinct areas that comprise each administrative neighborhood. The definition of a minimum population size allowed less variability between the local neighborhoods in terms of their population contingent.

Although we did not define an upper limit to the population size at the local neighborhood, we avoided the strategy of partitioning the administrative neighborhoods in a way that would create units with higher population size. We consider that this limit should vary according to the objectives, strategies and actions related to the phenomenon or health event of interest. The social characteristics as social cohesion, cultural habits and collective values, for example, should be identified or not depending on the scale, the spatial units and, consequently, on the population size delimited. In a recent study that compares different ways of delimiting neighborhoods, the authors concluded that the size and composition of the neighborhoods may be different in different parts of a study area [47].

A census tract is classified as showing irregular occupation when the occupied areas were originally invaded (by squatting), with no prior order in the mode of occupation, and where the residents do not own their homes (although they later may obtain adverse possession or property deeds). Census tracts contiguous to the *favelas *have suffered a process of steady real estate devaluation, and in many cases, from the socioeconomic point of view, they are very similar to the irregular tracts or *favelas *themselves [48-50].

As shown in Figure 2 and Figure 3, the composition of local neighborhoods in relation to areas with irregular tracts (*favelas*) proved quite satisfactory. Some areas of the city's South Side show situations of major contrasts (situation D and E, for example), with abrupt changes in the residential and socioeconomic profile between different census tracts (Figure 3). In these cases, the proposed method achieved good discrimination between these different patterns. Other areas (situation A and B, for example) around the irregular census tracts (*favelas*) showed census tracts with regular occupation, but with a population having a similar socioeconomic profile, with no change in the population pattern. In these situations, in which the groups display the same socioeconomic pattern, it was possible to identify this similarity (continuity), since they constituted the same local neighborhood (Figure 2). As one shifts to the city's West Side (Zona Oeste), this situation becomes common, i.e., with fewer heavily contrasting areas.

Another example of the capacity to discriminate between different socioeconomic patterns in local neighborhoods was the partitioning of the Rocinha *favela*, an administrative neighborhood with approximately 60,000 inhabitants, into seven distinct local neighborhoods. Figure 3 shows the local neighborhoods' boundaries and the differences in the pattern of residential occupation that reflect the different socioeconomic conditions captured by the indicators used in defining the local neighborhoods. Figure 4 shows the profile of indicators characterizing each of the seven local neighborhoods demarcated in Rocinha. Although the entire area of the administrative neighborhood consists of irregular tracts, Figure 3 and Figure 4 illustrate how it was possible to differentiate between areas with older occupation, which have a better street layout allowing easier access, and those with more recent occupation, with worse sanitation and more precarious and less vertical housing, that is, distinct areas whose residents show different income and schooling patterns and a different concentration of children and total occupants in the households.

Although Figures 5 to 8 show a simplified distribution of the indicators in only four categories, it illustrates the importance of each of the four socioeconomic indicators in demarcating the local neighborhoods. Each single indicator's contribution is limited in terms of discriminating the different internal compositions of the administrative neighborhoods. However, combined use of the four indicators optimizes this capacity, with the joint configuration producing the partitioning that allowed distinguishing between the 794 local neighborhoods.

The income and schooling indicators allowed capturing different socioeconomic dimensions that exert distinct impacts on health conditions [51], so both should be considered in studies on social inequalities in health. The mean number of persons per household, an indicator of household crowding, is essential to capture the population's living conditions. Due to changes in the variables studied in the population census, there is no longer information on the mean number of household residents per room, an indicator traditionally used to characterize urban occupation [46,47], since the households comprising the areas with the best social conditions have low resident-per-room density, while those with the worst conditions show high density. Thus, the mean number of inhabitants per household, although presenting a narrower range, proved adequate for differentiating between household density patterns in the various areas. Finally, the population's age composition, captured by the proportion of inhabitants from zero to four years of age, is an important demographic indicator with great capacity to characterize different social profiles in relation to the population turnover and growth in urban areas, especially in developing countries like Brazil [17].

There is general consensus in the literature that neighborhood refers to geographic units of a limited size, with relative internal demographic and residential homogeneity, as well as some level of social interaction and symbolic meaning for residents. Despite the growth in the literature exploring neighborhood effects on health, little attention has been paid to criteria and methods for demarcation of neighborhoods. The capacity to differentiate socio-spatial inequalities, demonstrated by the partitioning of local neighborhoods grasped by means of the proposed method, will allow performing new studies on the effects of the population's living conditions on health.

The process of identifying the boundaries of a neighborhood depends in part on the definition of the neighborhood that is appropriate to a particular planning initiative or study i.e., social, physical, or political. Consequently, there is no one ideal way of defining a neighborhood and its spatial boundaries [47].

The effect of neighborhood conditions should be looked at using several different ways to define boundary neighborhood identification: administrative (i.e. census tracts, ZIP codes), political (i.e. defined by associations, organizations), recognized (i.e. resident perception map, cognitive mapping) and created (i.e. school neighborhoods) [33,51-53]. In a recent series of North American and British studies, various political-administrative units of analysis were referred to generically as "neighborhoods" [21]. This is the main way that it has been used to study the effect of neighborhood conditions on health. There is little in the public health literature suggesting that alternative methods for delineating neighborhood boundaries have been attempted [28].

Weiss and colleagues (IMPACT study) utilized a multi-step neighborhood definition process including development of census block group maps, review of land use and census tract data, field visits and street-level observations. Defined neighborhoods (36 - 3 in each of 12 NYC communities) range from 1 to 8 census block groups, with populations ranging from 2252 to 11,503 (mean = 5320). Authors inform that the use of observation as part of the boundary definition process facilitates the identification and grouping of census block groups, having attributes consistent with the concept of "neighborhood" and with the study objectives. However, considering time and funding perspective, they concluded that, although subjectivity cannot be eliminated, neighborhoods defined this way can be compared to block group combinations identified by cluster analyses of census data [54].

The researchers of the Project on Human Development in Chicago Neighborhoods (PHDCN) defined neighborhoods with a method of census tracts direct aggregation, considering contiguity and some sociodemographic indicators from census (i.e. racial-ethnic composition). Sampson and colleagues collapsed 847 census tracts in the city of Chicago to form 343 neighborhood clusters - an ecological unit of about 8,000 people, large enough to approximate local neighborhoods; respectful of geographic boundaries and knowledge of Chicago's neighborhoods [55].

Despite we did not evaluate the symbolic significance to residents, local neighborhoods demarcated were conceptualized based on similar goal to of the two studies described above [54,55], looking for geographic units of limited size, with relative homogeneity in housing and population, as well as some level of social interaction. Some advantages were expected with this study's methodology because we used available databases, free TerraView software and easy tools to deal with visual overlapping analysis. These characteristics allow researchers to develop studies with fewer funding and to deal with complexities presented by large urban settlements as diverse as Rio de Janeiro city.

In Brazil, there was no study published using other neighborhood demarcation than political-administrative boundaries. Only one proposal of spatial partitioning using cluster analysis was published at 1996, by Carvalho et al. [44], applied in an island of Rio de Janeiro municipality.

It is hoped that the use of local neighborhood spatial units of analysis in studies on the properties of contextual characteristics, like those that have been developed in the PHDCN [29], can be implemented in many Brazilian cities. Socioeconomic characteristics of neighborhoods, like income, schooling, age composition, racial/ethnic composition, and indices of inequality, poverty, and affluence, are associated with various health outcomes [28], including self-rated health [56] and lifestyle [27]. Signs of physical disorder reflect the deterioration of urban space and are associated with worse health conditions [57,58].

We particularly hope to further the study of psychosocial characteristics in the local neighborhood context and their role in the determination of health outcomes. Characteristics of the social setting like cohesion and social control, establishment of networks, organizations, and prevalent lifestyle can promote or jeopardize health. The differential capacity of neighborhoods to reinforce the residents' common values and the maintenance of effective social control explain the variations in violence rates in Chicago that are not attributable only to aggregate individual demographic characteristics [55]. Collective efficacy (the combination of mutual trust and intention to intervene for the common good) acts as a mediator of the effects of socioeconomic stratification on violence. Informal social control and collective efficacy can also be generalized to a series of important objectives for the well-being of neighborhood populations [11].

In parallel with the study, another important step for the development of neighborhood and health approaches in Brazil, especially in Rio de Janeiro, is the enhancement of data georeferencing capacity for health events through precise localization of addresses in census tract and local neighborhood spatial units. Currently, most of the information published on health events only reaches the administrative neighborhood or administrative district level. Isolated initiatives require great effort for georeferencing at smaller levels, which limits the availability of health data to specific studies [19]. This situation should change in the coming years, since the National Census Bureau (IBGE) is consolidating a street registry comprising all the census tracts of municipalities with more than 100 thousand inhabitants and has announced that it will make the registry available shortly for use in a system to locate addresses by census tract [59].

The influence of social processes on health is increasingly clear, and it does not suffice to merely shape a population cluster if its spatial unit of analysis fails to capture the social processes taking place between a population and its place of residence.

When studying the properties of local neighborhood spatial units, one should not lose sight of their place on macro-determinant scales. As shown in Figure 10, according to the currently proposed method, population groups were clustered in local neighborhood spatial units that are nested in administrative neighborhoods. Administrative neighborhoods, in turn, are nested in a continuum of hierarchical levels up to global levels. The multiplicity of different levels can be relevant for some research questions. Super-groups represent division of the municipal territory in major groups of local neighborhoods that express other possibilities for aggregation in which spatial contiguity is not important. The specification of relevant levels for given studies is one of the theoretical definitions that precede data collection and statistical analyses [11]. The local neighborhood is thus only one of the scales, the one closest to the local level, but it is not always the most appropriate, and it is especially not the only one to contribute to the contextual effects on health [60].

We hope that it will be possible to evaluate the adequacy of local neighborhood spatial units proposed for health investigations in order to study different health events, such as violence, communicable diseases, and mortality.

## Final Remarks

We agree with Ana Diez-Roux et al [61] that Epidemiology is very sophisticated at measuring characteristics at the individual level, but not as sophisticated at measuring patterns in ecological sets. This seriously affects our capacity to examine contextual effects. In the current study, we present an approach that minimizes the problems related to residential heterogeneity between areas and maximizes the possibility to identify contextual characteristics permeating social processes within local neighborhoods.

Since the proposed method for demarcation of local neighborhoods is a structured method based on available data and open source computer programs and that can be easily reproduced in other cities, both in Brazil and abroad, we hope that it will allow progress on studies of intra-urban social differentials in the residential context and their implications for various health outcomes.

We emphasize that there is not just one way of demarcating neighborhoods. The proposed local neighborhood method is one of the possible ways of differentiating intra-urban space. Using this method, it was possible to construct spatial units that integrate populations with similar profiles and that are geographically proximate. This approach can be used and adapted to different constructs, depending on the study problem and underlying theoretical model. In this case, various parameters can be altered, like the minimum population size and the target indicators.

## Competing interests

The authors declare that they have no competing interests.

## Authors' contributions

SM Santos participated in the article's conceptualization and conducted the literature review, structured the database, analyzed and interpreted the compiled data, and wrote the article. D Chor and GL Werneck participated in the article's conceptualization and contributed to the analysis and interpretation of the results and helped write the article. All authors read and approved the final manuscript.
